# A cloud-based pipeline for analysis of FHIR and long-read data

**DOI:** 10.1093/bioadv/vbac095

**Published:** 2023-01-20

**Authors:** Tim Dunn, Erdal Cosgun

**Affiliations:** Computer Science and Engineering, University of Michigan, Ann Arbor, MI 48109, USA; Biomedical Platforms and Genomics, Microsoft Research, Redmond, WA 98052, USA

## Abstract

**Motivation:**

As genome sequencing becomes cheaper and more accurate, it is becoming increasingly viable to merge this data with electronic health information to inform clinical decisions.

**Results:**

In this work, we demonstrate a full pipeline for working with both PacBio sequencing data and clinical FHIR^®^ data, from initial data to tertiary analysis. The electronic health records are stored in FHIR^®^ (Fast Healthcare Interoperability Resource) format, the current leading standard for healthcare data exchange. For the genomic data, we perform variant calling on long-read PacBio HiFi data using Cromwell on Azure. Both data formats are parsed, processed and merged in a single scalable pipeline which securely performs tertiary analyses using cloud-based Jupyter notebooks. We include three example applications: exporting patient information to a database, clustering patients and performing a simple pharmacogenomic study.

**Availability and implementation:**

https://github.com/microsoft/genomicsnotebook/tree/main/fhirgenomics

**Supplementary information:**

Supplementary data are available at *Bioinformatics Advances* online.

## 1 Introduction

When visiting the doctor’s office for an annual physical, it’s typical to have your vitals taken. Weight, blood pressure and heart rate are all important measurements that can indicate impending health issues. While waiting in the lobby, it’s also common to fill out a short survey regarding sleep, smoking, drug and other lifestyle habits that may impact your health. Some doctors even recommend blood work—laboratory testing which measures cell counts and micronutrient levels—the results of which could indicate other less visible issues. This smattering of multi-modal information is then used by doctors to make informed decisions about lifestyle and medication changes that may improve your overall health. The more information a trained medical professional has available, the better recommendations they can make toward improving their patient’s health.

Soon, genomics data may routinely be used to complement this clinical data. The first ‘complete’ human genome was finished in 2000 at an estimated cost of $300 000 000 (Wetterstrand, 2021). Due to rapid improvements in sequencing technologies, however, this cost has sharply declined over the past two decades. Currently, whole genome sequencing costs around $700 per patient (Wetterstrand, 2021) and is usually reserved only for those suffering from cancer or rare genetic diseases. In just a few years, however, the cost will likely be low enough for routine sequencing of ordinary patients.

Not only can genome sequencing lead to earlier and more accurate genetic disease and cancer diagnoses, but it can also be used to predict individualized responses to medications and characterize the body’s internal micro-organisms and pathogens. For example, sequencing has been widely used to identify exact SARS-CoV-2 strains (Pater *et al.*, 2021) and analyzing the gut microbiome can lead to insights regarding overall well-being (Ji and Nielsen, 2015). Once sequencing costs have lowered, it will be possible to integrate genomic data with existing clinical data to provide a more comprehensive view of each and every patient. This data will, in turn, lead to a better understanding of patient health and disease—particularly with the help of machine learning.

Machine learning has caused immense scientific progress in recent years when applied to new domains such as text recognition, protein folding and nanopore sequencing basecalling (Jumper *et al.*, 2021; Rang *et al.*, 2018; Wang *et al.*, 2012). Unfortunately, there are a number of legal, practical, and ethical concerns preventing the immediate use of machine learning for diagnosing patients (Char *et al.*, 2020; Food, Drug Administration *et al.*, 2019; Kelly *et al.*, 2019). In the inevitable case of false positives and false negatives, how can we perform root cause analysis or ensure that the same mis-diagnoses won’t happen again? In many cases, we can’t. Machine learning can be used, however, to find correlations between genetic alterations and clinical observations, which can be used to guide further scientific research. Used properly, machine learning can be a tool for discovery, accelerating our progress in understanding genetic diseases and even lead to advances in medication and gene therapy. In this work, we present a scalable and secure proof-of-concept pipeline for combining clinical and genomic data in the cloud and demonstrate several possible use cases.

### 1.1 Clinical data

#### 1.1.1 FHIR^®^: Fast Healthcare Interoperability Resource Format

Clinical data can come in the form of numbers, raw text, images, or even 3D scans. Despite the inherent diversity of this data, it must be stored in a consistent digital format that allows for easy and efficient exchange between hospitals, laboratories and data centers. The ‘Fast Healthcare Interoperability Resource’ (FHIR^®^) format is the current leading standard for healthcare data exchange (Bender and Sartipi, 2013). Each chunk of FHIR^®^ data is an instance of one of 140 pre-defined resources, represented in XML, JSON or RDF format. The framework was designed to be broad and extensible, covering clinical healthcare, clinical trials, organization administration and finances. Data are commonly hosted on a secure server and accessed from an FHIR^®^ RESTful API that ensures secure and efficient querying of patient data.

#### 1.1.2 Synthea

**Fig. 1.**
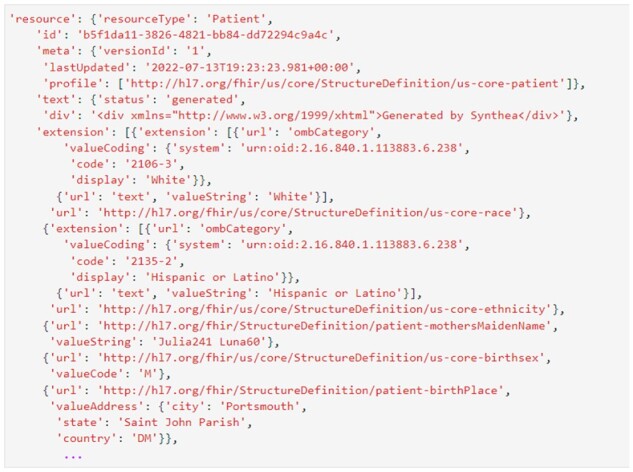
Example synthetic FHIR^®^ data, generated with Synthea

Synthea is a widely-used open-source tool for generating realistic (but synthetic) patient data in FHIR^®^ format (Walonoski *et al.*, 2018). This enables researchers to work with realistic clinical datasets without worrying about any of the legal, ethical or security concerns that would accompany working with real patient data. Figure 1 shows a snippet of realistic patient data generated with Synthea.

### 1.2 Genomics data

#### 1.2.1 Sequencing technologies

Illumina short-read sequencing remains the dominant technology for genome sequencing today. For years, it has successfully provided relatively low cost and massively-parallel short-read sequencing. Recently, however, newer long-read technologies have begun to prove competitive. Illumina short reads of several hundred bases typically achieve around 99.9% accuracy (Fox *et al.*, 2014). The newer and less mature long-read sequencing technologies such as Pacific Biosciences SMRT (Rhoads and Au, 2015) and Oxford Nanopore (Jain *et al.*, 2016) haven’t achieved similar accuracy results until very recently. These newer technologies can easily achieve average read lengths of over 10 000 bases (Jain *et al.*, 2016). This greatly aids in assembling the human genome in complex or repetitive genomic regions, and PacBio HiFi reads were instrumental in the ‘Telomere-to-Telomere’ consortium completing the first truly complete human genome in 2021 (Nurk *et al.*, 2022).

#### 1.2.2 Variant calling

**Fig. 2.**
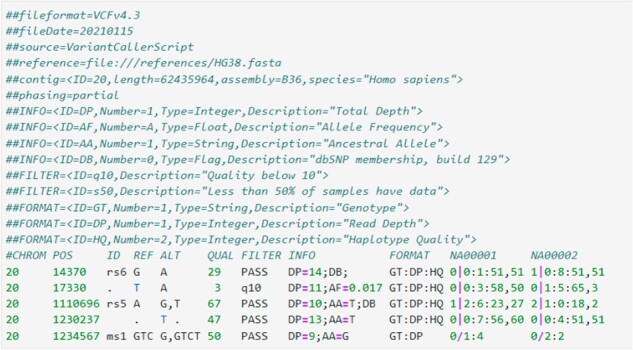
Example VCF data, including both the file header and data. The first eight tab-separated fields refer to a specific variant, and the remaining fields store information about that variant as it pertains to each sample

Since two human genomes are 99.9% identical (NHGRI, 2018), the end goal of most DNA sequencing efforts is to identify the differences between an individual’s DNA and a standard reference sequence. This problem is known as ‘variant calling’. These small changes in DNA can be in the form of single-nucleotide polymorphisms (abbreviated SNPs, A→G), insertions (A→ATT), deletions (AGC→A) or structural variants (in which large segments of DNA are inserted or deleted). In this work, we focus on small variants, which include SNPs and insertions or deletions shorter than 50 bases. These variants are stored in ‘Variant Call Format’ (VCF), which notes the ‘reference’ and actual (‘alternate’) observed DNA sequence. Figure 2 shows an example. Databases of known mutations and their functional consequences (if any) on patient health are used to identify important mutations (Hutter and Zenklusen, 2018).

### 1.3 Cloud Frameworks

#### 1.3.1 FHIR^®^ integration

All of the major cloud providers have their own implementation of an FHIR server that can readily be used with other cloud services. Amazon supports ‘FHIR Works’ on Amazon Web Services (AWS), Microsoft supports an ‘Azure API for FHIR’ on Azure, Google supports a ‘Cloud Healthcare API’ on Google Cloud and IBM supports an ‘IBM FHIR Server’ on the IBM Cloud (AWS Labs, 2022; Google Cloud, 2022; IBM, 2022; Microsoft, 2022c). There are numerous other open- and closed-source standalone FHIR server implementations as well, such as HAPI FHIR (Agnew, 2022). For our purposes, any of the FHIR server implementations that integrate easily with Cromwell and a major cloud provider would work. We selected Microsoft Azure and the ‘Azure API for FHIR’ simply because we had reduced-cost access to Azure Cloud computing resources.

#### 1.3.2 Bioinformatics using Cromwell

Cromwell is an open-source workflow management system designed by the Broad Institute for performing bioinformatics at scale (The Broad Institute, 2022). Cromwell can be configured to run with a Google Cloud backend through the Google Genomics Pipelines API, an AWS backend using AWS Batch, or an Azure backend using the ‘Cromwell on Azure’ project (Microsoft, 2022b; The Broad Institute, 2019). As mentioned in the previous section, we selected the Microsoft Azure backend. PacBio has released a public workflow for running a human whole genome sequencing (WGS) pipeline using ‘Cromwell on Azure’ (Pacific Biosciences, 2022). It begins with unaligned or aligned reads (in FASTQ or BAM format, respectively), and determines large scale structural variants using pbsv (Wenger *et al.*, 2019) and hifiasm (Cheng *et al.*, 2021). Reads are phased using WhatsHap (Patterson *et al.*, 2015) and small variants are called in VCF format with DeepVariant (Shafin *et al.*, 2021).

### 1.4 Related work

Previous works in merging clinical and genomic information have primarily focused on extending the FHIR^®^ implementation to include genomic data. Earlier efforts such as ‘SMART on FHIR Genomics’ were influential in envisioning the design of such a standard (Alterovitz *et al.*, 2015). A more recent project by the Electronic Medical Records and Genomics (eMERGE) Network developed a new standard format based on HL7^®^ FHIR^®^ to represent clinical genomics results (Murugan *et al.*, 2021).

There has been significant progress within HL7^®^ to adopt a standard format for sharing genomic data as well. In particular, HL7^®^ has organized a Clinical Genomics Work Group to tackle this effort (Health Level Seven International, 2022). The current version of the HL7^®^ FHIR^®^ specification includes a ‘Genomics Implementation Guidance’ page, which is currently in the ‘Trial Use’ phase of development, with a Maturity Level of only 1 (on a scale from 0 to 5). Over the past several years, the HL7’s recommendations for storing genomic data have shifted and evolved rapidly due to fast-paced technological advancements and learnings from practical experience. Regardless of whether the current standard is to store data in an Observation, Sequence, Observation-genetics, MolecularSequence or Variant resource, the format is not yet mature and there are no guarantees of long-term stability with regards to the current format. To avoid this problem, we convert the contents of stable FHIR^®^ resource implementations to a tabular format prior to merging with genetic information.

Although several previous works explore merging clinical and genomic patient data, they primarily focus on the release of large datasets of cancer patients. Project GENIE is the largest of such efforts, resulting in more than 100 000 sequenced patients from cancer centers worldwide (AACR Project Genie Consortium *et al.*, 2017). Despite the availability of merged clinical and genomic data—at least for cancer patients—there is no publicly available standard pipeline for merging the two data modalities. This work presents such a pipeline for secure and scalable merging of clinical and genomic data using cloud resources.

## 2 Methods

**Fig. 3.**
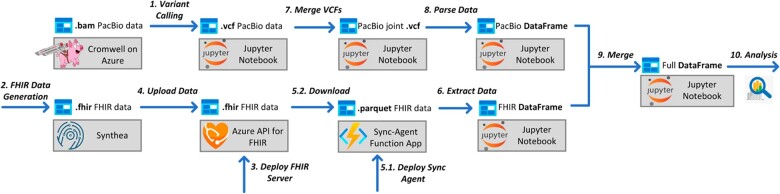
Overview of data processing pipeline, common to all demonstrated applications

Figure 3 shows an overview of our data processing pipeline, which was shared for all three of our example applications. This pipeline processes the PacBio and FHIR^®^ data prior to merging them into a single DataFrame for further analyses. In the following two sections, we first describe our treatment of the PacBio genomics data, and then our methods for dealing with the synthetic FHIR^®^ data.

### 2.1 Genomics data

For our genomics data, the first step was variant calling using Cromwell on Azure. A deployment script available from Microsoft’s Cromwell on Azure repository (Microsoft, 2022b) was first downloaded and executed to initialize the workflow environment. The GCA_000001405.15_GRCh38_no_alt_analysis_set.fasta reference genome was downloaded from the NCBI (Pruitt *et al.*, 2005) database, and a BED file containing tandem repeat regions to exclude was downloaded from the pbsv repository (Wenger *et al.*, 2019). In order to demonstrate the Cromwell workflow, we used the GIAB (‘Genome in a Bottle’) PacBio sequencing data for human sample HG002 (Zook *et al.*, 2016). We use sample VCF data from a larger dataset for all downstream processing. Additionally, we made several modifications to PacBio’s default human WGS workflow configuration (Pacific Biosciences, 2022). These changes consist of several bugfixes, which have now been merged into their official repository, and removing the tandem-genotypes step for simplicity.

Once the variants were called using Cromwell on Azure, we used BCFTools (Danecek *et al.*, 2021) to normalize the variant representation and split sites with multiple alleles. Normalizing involves left-aligning insertions and deletions (INDELs) and splitting multi-nucleotide polymorphisms in to SNPs (Danecek *et al.*, 2021). Splitting multi-allelic sites were necessary because in order to convert the VCF to TSV format, there must be a constant number of fields (columns) per entry (genomic position). By splitting entries with multiple possible variants into multiple entries—each with a single variant—we ensured that fields such as allele frequency are represented with a fixed number of columns.

Next, we performed linkage disequilibrium pruning to remove variants calls with high covariance, and ensure that most remaining variants have a fairly high degree of independence from one another. At this point, we used BCFTools to merge VCF files from all patients into a single VCF file, and extracted select fields into a TSV file. Since our input files were VCF, and not GVCF, they did not contain information regarding the quality of reference calls for non-variant positions. To deal with this, we assumed all missing entries to be a reference call of average quality and depth for that sample, and imputed the genotype and phred likelihoods under this assumption. As a final step, the TSV was loaded into a Pandas DataFrame and transposed so that each row corresponds to a patient, and the columns are the relevant genomic information: the genotype, allele frequency, depth and phred likelihood scores for a select group of variants.

### 2.2 Clinical data

**Fig. 4.**
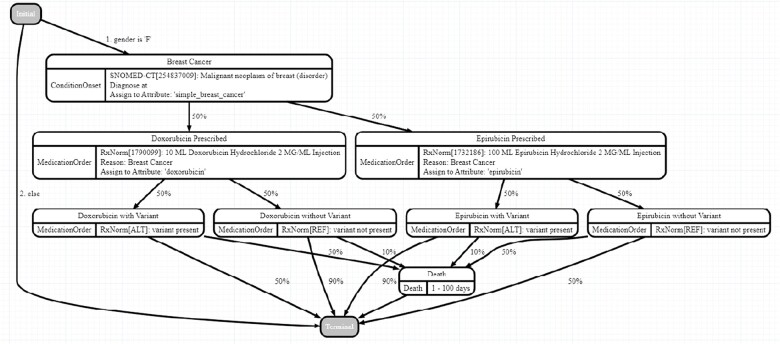
Custom Synthea module for modeling breast cancer treatments and outcomes

Using Synthea, we generated a synthetic dataset with the same number of patients as included in our sample VCF files. For our data exportation and patient clustering applications, we simply used Synthea with the default parameters to generate a typical patient population. For the breast cancer study, however, we added a custom module to model the simplified progression of breast cancer in a cohort of affected patients. This module is shown in Figure 4.

Combined with the Synthea flags -g F -a 30-90, this module generates an exclusively adult female dataset of breast cancer patients, who are treated with one of two medications: Doxorubicin or Epirubicin. Patient survival rates, assumed to be 50% if left untreated, depend on a combination of medication and presence of a specific variant. For patients without the variant, survival rates can be improved to 90% by prescribing Doxorubicin. For patients with the variant, the same survival rate is instead achieved by prescribing Epirubicin. With all other groups, the survival rate remains unchanged at 50%. This relationship which we’ve embedded into our synthetic clinical data generation module should be discoverable through downstream analyses, provided our patient cohort is large enough.

In a real clinical setting, patient records will be stored in FHIR^®^ format on a server, where they can be queried by clinicians. To model this setup, we deploy an FHIR^®^ server using the Azure API for FHIR, and transfer our generated Synthea data to the server using the REST API. Using the FHIR bulk import and export functionality would be a viable alternative (and a potential direction for future work). We found that performing individual requests allowed us to transfer fewer FHIR resources in total, and achieved sufficient throughput.

In order to perform data science applications with FHIR^®^ data, we first need to convert the hierarchical JSON data into tabular format. An existing open-source tool called the ‘FHIR to Synapse Sync Agent’ solves this problem by downloading each FHIR^®^ resource from a server and converting it to tabular Parquet format (Microsoft, 2022d). Parquet files store the same information as ordinary CSV (‘comma-separated values’) or TSV (‘tab-separated values’) files, but in a more efficient compressed manner. Data are stored in column-major order, which allows compression algorithms such as run length encoding, dictionary encoding or delta encoding to be applied per column depending upon each column’s data format and values (Ivanov and Pergolesi, 2020). It is important to note that although Parquet is a tabular format, it is still able to store unstructured or nested fields present in the original FHIR^®^ resource by encoding them as JSON strings.

**Fig. 5.**
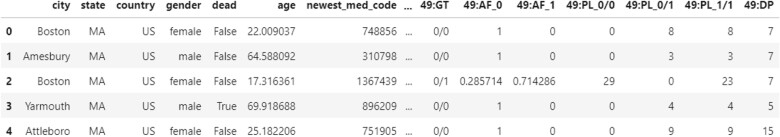
Final Pandas DataFrame of merged FHIR^®^ and PacBio data

Once downloaded, we parse the Parquet FHIR^®^ data to retrieve relevant information and load it into a Pandas DataFrame (McKinney, 2011). Although Parquet is already a tabular format, the converted data contains extraneous information which must be discarded, and any useful information stored within a JSON string must be extracted. Making matters more difficult, FHIR^®^ data pertaining to a single patient is stored in multiple resource types. These files (such as Patient, Medication, or Condition resources) must be parsed separately and the records associated with one another using the patient’s unique Medical Record Number (MRN). We are able to map information from multiple resources of the same type to a single patient by adding another column to the DataFrame for each resource instance. In the end, each row stores data for a single patient, and the numerous columns contain all desired information about that patient, extracted from the Parquet files. At this point, the FHIR^®^ and PacBio data can be merged together, as shown in Figure 5.

## 3 Results

After merging these two data modalities, we explore three example use cases for such data: exporting the information to a database, clustering patients and studying a cohort of breast cancer patients.

### 3.1 Application #1: export to database

The first such application that we explored was the simplest: exporting the FHIR^®^ and PacBio data to a database for further analyses. We selected Azure Synapse Analytics as our database platform because it ensures scalability and reliability. After converting our FHIR^®^ and PacBio DataFrames to Parquet format, we directly import them into Azure Synapse. From there, we can perform joint queries on the two datasets. This application is demonstrated in the Supplementary Notebook1-data-export.ipynb.

### 3.2 Application #2: patient clustering

The second application we demonstrated was an exploratory clustering of our patient dataset using three different clustering methods. Firstly, K-means++: an iterative approach which updates cluster centroid locations to minimize total inertia and uses repeated random initializations (Arthur and Vassilvitskii, 2007). Inertia is the average squared distance from each data point to the center of its labeled cluster. Secondly, we used DBSCAN: a recursive approach that builds clusters from high-density areas containing ‘core samples’ (Ester *et al.*, 1996). Lastly, Spectral Clustering, which performs a low-dimensional embedding of the samples’ affinity matrix prior to clustering (Stella and Shi, 2003).

**Fig. 6.**
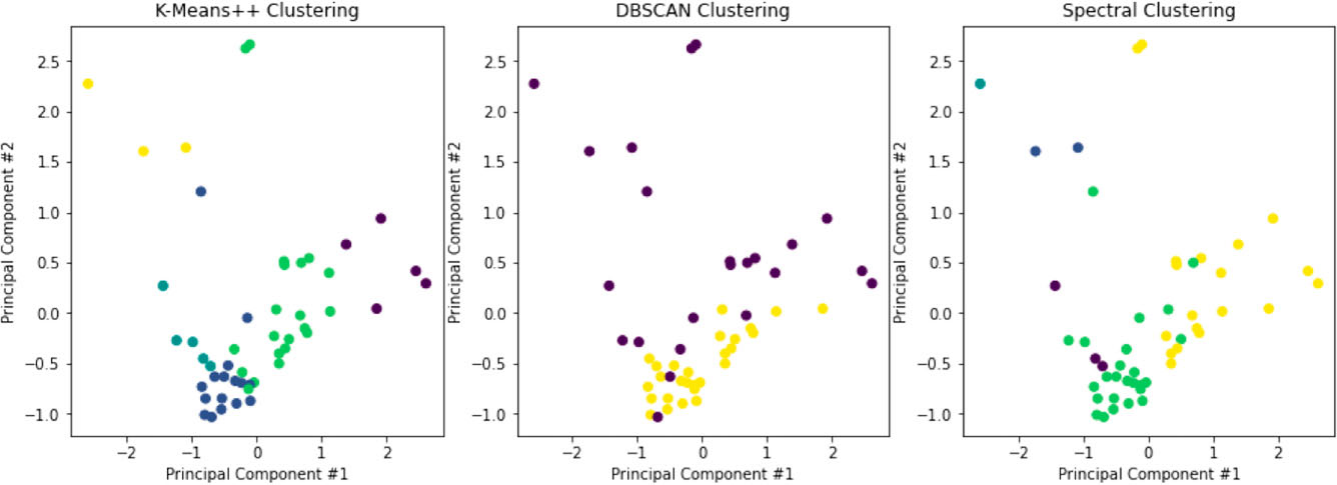
Patient clusters resulting from several different clustering algorithms

Both K-means++ and Spectral Clustering require specifying the final number of clusters a priori. To select a reasonable number of clusters we used the ‘elbow method’, which consists of plotting the number of clusters versus total inertia. As the number of clusters increases, the cluster inertia will always decrease, but once the data have already been clustered fairly well, there will be less benefit in introducing additional clusters. This shows up on an ‘elbow plot’ as a bend toward the horizontal, which we found occurs at *n *=* *5. After selecting *n* for K-means++ and Spectral Clustering, Figure 6 shows the resulting clusterings. Each point represents a single patient, and each cluster of patients is colored uniformly.

**Fig. 7.**
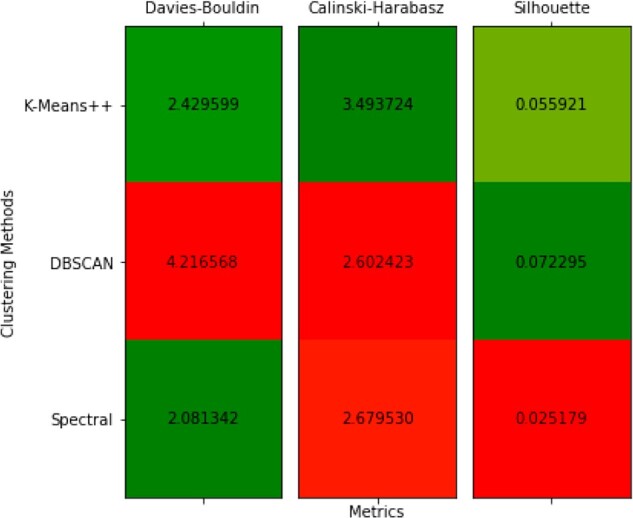
Evaluation of clustering methods using several different metrics

We evaluated these three clustering methods using the Davies–Bouldin Index (Davies and Bouldin, 1979), the Calinski–Harabsz Index (Caliński and Harabasz, 1974) and the Silhouette Coefficient (Rousseeuw, 1987). Unlike the other two metrics, for the Davies–Bouldin Index a lower score is better. These three evaluation metrics were selected because they all do not require knowledge of some ground truth clustering of samples into classes. Since we are simply trying to discover similarities between patients, no ground truth is available. As Figure 7 shows, K-Means++ selected the best clustering overall. This application and evaluation is included in the Supplementary Notebook2-clustering.ipynb.

### 3.3 Application #3: breast cancer study

**Fig. 8.**
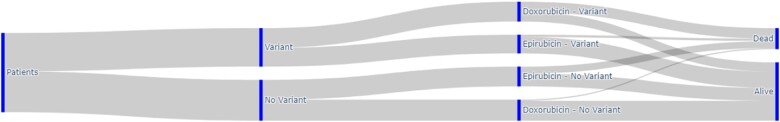
Sankey diagram showing distribution of patient treatments and outcomes

The final application we demonstrated was a basic pharmacogenomic study to determine the effect of two different medication treatments on breast cancer patients’ survival rates. As described in Section 2.2, a custom Synthea module was designed which modeled the fact that outcomes were only improved beyond the base 50% survival rate for patients who were prescribed Epirubicin and had a particular genetic variant, and for patients who were prescribed Doxorubicin and did not have the genetic variant. Figure 8 shows a breakdown of patient variant presence, treatment type, and survival.

We performed a one-sided z-test with a *P*-value of 0.01 to investigate whether patients in each of the four groups (all combinations of with/without variant and Doxorubicin/Epirubicin) had improved survival rates over the expected outcome for untreated patients (a 50% survival rate). As expected, we found a significant increase in survival rates for patients without the variant who were prescribed Doxorubicin (*P *<* *10^−8^), and for patients with the variant who were prescribed Epirubicin (*P *<* *10^−5^). For patients without the variant who were prescribed Epirubicin (*P *=* *0.109) and patients with the variant who were prescribied Doxorubicin (*P *=* *0.5932), we did not find a significant improvement in outcomes, as expected. This application is included in the Supplementary Notebook3-pharmacogenomics-confidential.ipynb. The custom Synthea module definition is included in Supplementary File3-simple-breast-cancer-module.json.

### 3.4 Confidentiality

When dealing with patient health information, ensuring confidentiality and data integrity is paramount. In order to ensure that all data processing is secure, our pipeline works within a Jupyter notebook hosted on an Azure ‘Confidential Compute’ virtual machine (Perez and Granger, 2015). Results are then made available on a local machine using SSH tunneling. These virtual machines have security features such as secure boot, a virtual trusted platform module (vTPM), boot integrity monitoring, and virtualization-based security (Microsoft, 2022a). Together, these features protect against persistent or advanced threats such as rootkits or bootkits, and ensure that the virtual machine has booted into a trusted environment as expected. Virtualization provides further security by isolating memory address spaces to remove any possibility of memory cross-contamination. Additionally, depending upon the underlying hardware, compute instances will include either Intel Software Guard Extensions (Intel SGX) or AMD Secure Encrypted Virtualization (SEV-SNP) support.

## 4 Discussion

One of the most unique aspects of this study is the analysis of long-read sequencing data together with clinical data for the first time. The outputs of the study will be an important use-case for researchers who are working on genetic data analysis. It is very important to acquire the necessary knowledge for the creation of decision support systems. We envision that eventually analyses such as our pharmacogenomic study will become commonplace, an automated analysis that occurs in real-time. This would provide clinicians with the ability to recommend prescriptions and treatments which are specifically tailored to the genetic makeup of each patient, based on the responses of similar individuals to each possible treatment. These types of research studies will also form the basis of data science models that are likely to be used in the near future.

The bulk of our pipeline is responsible for processing genomic and clinical data in a manner that ultimately results in a tabular format containing a row for each patient and a column for each feature. Machine learning on tabular data works well with a restricted set of features, but does not scale well as the number of columns increases due to the ‘curse of dimensionality’ (Verleysen and François, 2005). Moreover, some information is not easily represented in tabular format without loss of information or extreme data sparsity. For example, storing the medications taken by each patient would require either a Boolean column for each possible medication, or categorical columns storing the last *n* medications taken by each patient. Most patients may only take one or two medications, but the most heavily medicated patient may take dozens.

In order for machine learning to make complex clinical decisions, such tools will eventually need to be able aggregate information from data in many different formats. One possible solution would be an ensemble of classifiers which each work with a different data modality such as tables, graphs, images, and natural language. Multimodal machine learning is an active area of current research with great potential in the healthcare field (Acosta *et al.*, 2022).

Although we demonstrate variant calling using Cromwell on Azure for a public single patient dataset, the current inputs to our pipeline are a larger set of sample VCFs resulting from PacBio’s human whole genome sequencing workflow. We do not currently have access to the original reads used to generate these VCFs. In future work, we would like to run a modified Cromwell workflow which outputs results in GVCF format. Unlike VCF files, GVCF files contain variant calling information about all (including reference/non-variant) positions on the genome, grouped into blocks of configurable size. VCF files only report confidently called variants. In this work, we impute reference quality and depth scores using the genome-wide average, akin to a GVCF with an exceptionally large block size. Transforming our pipeline to use GVCF inputs would simultaneously simplify post-processing of variants and improve the quality of all data regarding reference calls.

## Supplementary Material

vbac095_Supplementary_Data

## Data Availability

The long-read sequencing data underlying this article cannot be shared publicly to preserve the privacy of individuals in our patient cohort.The synthetic FHIR data underlying this article can be re-generated using the software available in the article's online supplementary material.
